# Development of an enzyme-linked immunosorbent assay for diagnosis of human herpesvirus-7 infection

**DOI:** 10.1590/0037-8682-0181-2019

**Published:** 2020-03-16

**Authors:** Ana Lia Pradella Puglia, Murilo de Freitas Peigo, Fernando Russo Costa Bomfim, Ronaldo Luis Thomasini

**Affiliations:** 1 Instituto Butantan, Laboratório de Imunologia Viral, São Paulo, SP, Brasil.; 2 Fundação Hermínio Ometto, Centro Universitário, Laboratório de Patologia Clínica, Araras, SP, Brasil.; 3 Fundação Hermínio Ometto, Centro Universitário, Departamento de Biologia Molecular, Araras, SP, Brasil.; 4 Universidade Federal dos Vales do Jequinhonha e Mucuri, Faculdade de Medicina de Diamantina, Núcleo de Estudos de Patologias Infecciosas e Inflamatórias, Programa Multi-Cêntrico de Pós-Graduação em Ciências Fisiológicas, Diamantina, MG, Brasil.

**Keywords:** ELISA, IFA, HHV-7, Serological Assay

## Abstract

**INTRODUCTION:**

Human herpesvirus (HHV)-7 establishes a latent infection during the lifetime of the host and can reactivate after the primary infection, leading to lytic replication in immunosuppressed patients.

**METHODS:**

This study aimed to develop an enzyme-linked immunosorbent assay (ELISA) to identify HHV-7 serum antibodies and compare its performance with that of an indirect immunofluorescence assay (IFA).

**RESULTS:**

Serum samples (n=102) were tested by IgG-IFA and by ELISA. IFA and ELISA showed IgG-positive results in 77 and 73 samples, respectively. Qualitative concordance of 96% was demonstrated between the two techniques.

**CONCLUSIONS:**

ELISA may be useful to diagnose HHV-7 infection.

Human herpesvirus (HHV)-6, HHV-7, and human cytomegalovirus (CMV) are included in the subfamily *Betaherpesvirinae* of the *Beta-Herpesviridae* (DNA viruses). HHV-7 was first isolated by Frenkel et al.^1^from activated CD4+ peripheral blood T cells obtained from a healthy adult. 

The HHV-7 virus is ubiquitous and has a seroprevalence rate of 93.3% in northern Brazil^2^. HHV-7 infections in early childhood lead to common febrile infectious syndromes, known as exanthema *subitum* and roseola. After the primary infection, the virus remains in the host, can replicate in the salivary glands and be shed in the saliva, and remains latent in CD4+ T cells^3^.

Similar to other herpesviruses, HHV-6 and HHV-7 remain latent for a long term, and can reactivate in organ transplant recipients receiving immunosuppressive drug therapy. Reactivation during the post-transplantation period may result in the development of symptoms. Most HHV-6 (variants A and B) and HHV-7 infections in transplant recipients have asymptomatic features, and symptomatic disease is not frequently reported after organ transplant. Thus, routine laboratory testing for HHV-6 and HHV-7 DNAemia is not advised for prophylaxis in asymptomatic patients or for preemptive therapy^4^.

The immunomodulatory properties of HHV-6 and HHV-7 are related to increased opportunistic infections, allograft rejection, and an association between HHV-6 or HHV-7 reactivation, leading to an increased risk of CMV disease among adult kidney and liver recipients^5^.

HHV-6 reactivation after hematopoietic stem cell transplantation (HSCT) causes significant morbidity and mortality; however, scarce information is available for HHV-7 reactivation after HSCT. Few studies of post-transplant HHV-6 and HHV-7 infection have been done in children, although a recent study claims that HHV-6 and HHV-7 often cause infections after pediatric HSCT^6^.

HHV-7 is often diagnosed in adult liver transplant patients^7^ and bone marrow transplant recipients, which is associated with acute graft-versus-host disease and reduced survival time^8^. The clinical features of HHV-7 infection post-transplantation are not completely defined. However, it may have features similar to HHV-6, including a roseola-like illness, and less frequently, fever with simultaneous detection of HHV-7 and CMV in blood samples^9^.

Studies concerning the immune response against HHV-7 in transplant recipients and immunocompetent hosts may contribute to understanding the immunopathological features of HHV-7 infections.

Presence of antibodies to HHV-6 and 7 could indicate prior infection, and perhaps presence of the virus in the patient. Although serum neutralization and immunofluorescent assay (IFA) have been used to detect the presence of serum antibodies, a rapid, specific, and sensitive assay for the detection of HHV-6 and 7-specific antibodies is needed.

In this study, we evaluated the use of an enzyme-linked immunosorbent assay (IgG-ELISA) in comparison to IFA for the diagnosis of HHV-7 infection. Serum samples (n=102) were obtained from healthy adult individuals, including positive (n=77) and negative (n=25) subjects pre-tested for IgG against HHV-7 by IFA in sera. Among the total number of HHV-7 negative sera, four HHV-6 positive sera were included to investigate cross-reactivity. The protocol was designed in accordance with the requirements for research involving human subjects in Brazil (Approved by the Institutional Ethics Committee of Fundação Herminio Ometto).

HHV-7 isolated from a healthy donor was used to infect cell culture for antigen preparation. Briefly, cord blood mononuclear cells (CBMCs) were purified using a density gradient (Ficoll-Hypaque, GE Healthcare Bio-Sciences AB, Uppsala, Sweden), washed with PBS (phosphate-buffered saline) and cultured at 37^°^C for 72 hours in RPMI-1640 medium (Cultilab, Campinas, Brazil) containing fetal bovine serum and phytohemagglutinin. Cultured CBMCs were inoculated with filtered saliva (0.22µm) containing the virus, and were monitored for cytopathic effects for 21 days. The specificity of HHV-7 isolation was confirmed by polymerase chain reaction (PCR) and by IFA using monoclonal antibodies against HHV-7 (KR-4, Advanced Biotechnologies Inc., Maryland, USA). Presence of HHV-6 was also determined by use of monoclonal antibodies against HHV-6A/B (Mab8533 and Mab8535, Chemicon International, Temecula, USA), and negative cultures for HHV-6 were used for antigen preparation.

In the Anti-IgG IFA, HHV-7 cells infected with virus were coated onto wells of immunofluorescence slides, air-dried, and fixed. The wells were covered with a serial dilution of patient sera (starting from a 1:10 dilution) and incubated for 1 h at 37^°^C. Slides were washed with PBS, treated with an anti-human IgG fluorescent conjugate (Biomèrieux Inc., Lyon, France), and incubated for 1 h at 37^°^C. The slides were then washed, mounted, and observed under a UV photo microscope (Leica DM2000, Wetzlar, Germany). The antibody titer was defined as the reciprocal of the serum dilution showing fluorescence. Samples that presented IFA titers greater than 1:10 were considered positive.

Subsequently, for Anti-IgG ELISA, approximately 1.8 x10^6^ HHV-7 infected cells were lysed by freezing-thawing followed by incubation for 1 h at 37^°^C with 0.05% of Triton-X. The lysate was then purified by centrifugation and frozen until further use. Polystyrenemicroplates (Tyco HealthCare-Kendall, MA, USA) were coated with HHV-7 antigen. The antigen was diluted 1:2 in the carbonate-bicarbonate buffer (pH 9.6), added into each well of the microplates, and then incubated overnight at 4-8ºC. The plates were washed five times with PBS and then blocked with 1% bovine serum albumin (BSA) for 4 h at 37^°^C. The plates were washed with PBS and considered ready for use. Subsequently, 50 µL samples (1:100 in PBS 1% BSA) were dispensed into microplate wells and incubated for 1 h at 37°C. Microplates were washed and 50 µL anti-human IgG-HRP conjugate (1:50 in PBS- 1% BSA) was added to wells. Plates were washed with PBS, and the reaction was treated with tetra-methyl-benzidine and 0.03% hydrogen peroxide for 30 minutes. The reaction was blocked with 50 µL of 1N H_2_SO_4_.

The absorbance of the wells was measured in a microplate reader at 450nm and samples that demonstrated an absorbance ratio > 1.1 (absorbance sample divided by cut-off index) were considered to be positive. A standard curve was constructed based on a serial ten-fold dilution of positive serum with an IFA titer of 1:2,560 (1:2,560 [dilution 1:1], 1:256 [dilution 1:10], 1:25.6 [dilution 1:100]), and a negative serum. 

A statistical analysis of categorical variables was performed by Fisher´s exact test or Chi-Squired test. Continuous variables were reported as the average, range, and standard deviation (SD) and with a Spearman´s Correlation test. ROC curve analysis was performed to calculate the cut-off index for discrimination between negative and positive samples. The significance level was set at 0.05. For statistical analysis of the data, the program GraphPad Prism for Windows version 5.0 was used.

The average absorbance value of the ELISA wells coated with BSA was 0.02 (range: 0.01 to 0.03; SD: 0.01). The average absorbance value of the negative control was 0.0164 (range: 0.011 to 0.0230; SD: 0.005). The standard curve revealed an R^2^=0.99 and the ROC curve analysis demonstrated a specificity of 100% and sensitivity of 94.8% (95% CI; 86.28% to 100.0%) with a cut-off index of 0.0470, where the IFA was considered as the gold standard (Figure 1).

**FIGURE 1:**
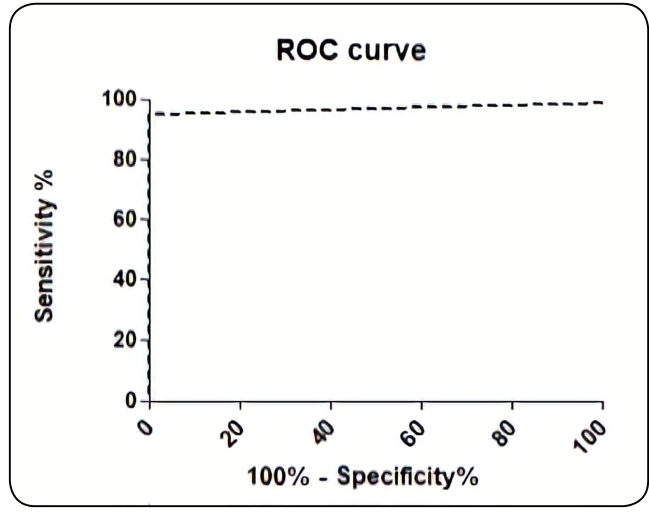
ROC curve of ELISA considering IFA as a gold standard.

In our results, positive IgG was detected in 77 samples (75.49%) by IFA and 73 samples (71.56%) by ELISA. Out of 77 HHV-7 positive samples by IFA, 73 (94.8%) were positive for IgG-HHV-7 by ELISA. Four positive samples for HHV-7 by IFA were negative for HHV-7 by ELISA. All HHV-7 negative samples by IFA were also negative for HHV-7 by ELISA. No HHV-6 positive samples presented cross-reactivity to HHV-7. The comparison of categorical variables (results categorized into ‘positive’ or ‘negative’) of the two techniques demonstrated concordance in 98 out of 102 total samples (96%) (p<0.001). However, the differences in continuous variables (absorbance on ELISA *vs.* log IFA titers) were not significant (p=NS; Spearman r= 0.1673) (Figure 2).

**FIGURE 2:**
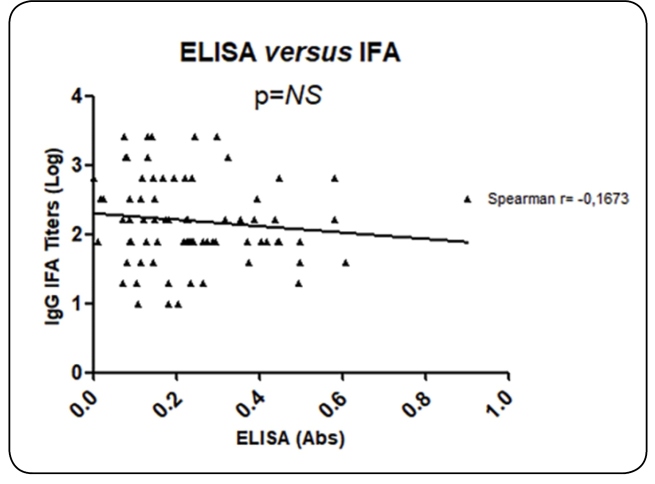
Correlation test for IgG antibody titers (IFA) and absorbance (ELISA) for HHV-7 antigens (Spearman´s Correlation test).

Diagnostic investigation for HHV-6 and HHV-7 includes serology, culture, antigenemia, immunohistochemistry, and PCR. Generally, whether these tests lack standardization, are inefficient at discriminating latent versus active infection or HHV-6A versus HHV-6B, and cross-reactivity between HHV-6 and HHV-7 may occur. Specific testing for HHV-7 is usually performed for research purposes, since no clinical syndromes associated with HHV-7 infection have been demonstrated. One of the major challenges in interpreting HHV-6 and HHV-7 tests is to determinate whether detection of the virus is related to an observed clinical symptom. The diagnosis of symptoms directly linked to HHV-6 or HHV-7 infection requires the exclusion of similar etiological agents^5^.

In this study, we found a HHV-7 seroprevalence rate of 75.49% by IFA and 71.56% by ELISA. Both ELISA and IFA results were correlated in 96% of cases whether the sample was categorized as ‘positive’ or ‘negative’ for IgG detection against HHV-7 antigens. However, no significant correlation was present between IgG titers (IFA) and absorbance (ELISA). A similar study that evaluated methods for detection of HHV-7 antibodies found a correlation of 83%^10^. Discordance between methods could be attributed to the use of different protocols for antigen preparation. Whole lymphocytes fixed on microscopic slides were used for IFA, and cell lysates (free of nuclei and cytoplasmic membranes) were used in ELISA. Detection of auto-antibodies against membranes and nuclei was, therefore, more likely in IFA than in ELISA. However, the lower sensitivity of ELISA compared to IFA cannot be disregarded. Subjective determination of IFA titers, difficult antigen standardization, and lack of standardized sera panels contribute to the lack of reliable and specific assays. The ELISA method presented in this study couldbe used as a preliminary test. However, it was necessary to perform a test by immunoblot assay using only the positive samples to identify discordant results between the IFA and ELISA tests. Furthermore, HHV-7 recombinant antigen has been reported^11^and could be used to standardize the ELISA method. Cross-reactivity between conventional antibodies against HHV-6 and HHV-7 must be considered. In addition, the use of IFA as a gold standard for HHV-7 serostatus must be reviewed.

In conclusion, the HHV-7 seroprevalence rate in our study was highly similar to that reported in other studies. ELISA and IFA results were correlated in most cases although non-concordant samples must be further analyzed. Thus, the ELISA method suggested in this study might be useful as a preliminary technique to diagnose HHV-7.
